# The Impact of Renin-Angiotensin System Blockade on Renal Outcomes and Mortality in Pre-Dialysis Patients with Advanced Chronic Kidney Disease

**DOI:** 10.1371/journal.pone.0170874

**Published:** 2017-01-25

**Authors:** Yun Jung Oh, Sun Moon Kim, Byung Chul Shin, Hyun Lee Kim, Jong Hoon Chung, Ae Jin Kim, Han Ro, Jae Hyun Chang, Hyun Hee Lee, Wookyung Chung, Chungsik Lee, Ji Yong Jung

**Affiliations:** 1 Division of Nephrology, Department of Internal Medicine, Cheju Halla General Hospital, Jeju, Korea; 2 Division of Nephrology, Department of Internal Medicine, Chungbuk National University Hospital, Cheongju, Korea; 3 Division of Nephrology, Department of Internal Medicine, Chosun University Hospital, Gwangju, Korea; 4 Division of Nephrology, Department of Internal Medicine, Gachon University Gil Medical Center, Incheon, Korea; 5 Division of Nephrology, Department of Internal Medicine, Gachon University School of Medicine, Incheon, Korea; The University of Tokyo, JAPAN

## Abstract

Renin-angiotensin-system (RAS) blockade is thought to slow renal progression in patients with chronic kidney disease (CKD). However, it remains uncertain if the habitual use of RAS inhibitors affects renal progression and outcomes in pre-dialysis patients with advanced CKD. In this multicenter retrospective cohort study, we identified 2,076 pre-dialysis patients with advanced CKD (stage 4 or 5) from a total of 33,722 CKD patients. RAS blockade users were paired with non-users for analyses using inverse probability of treatment-weighted (IPTW) and propensity score (PS) matching. The outcomes were renal death, all-cause mortality, hospitalization for hyperkalemia, and interactive factors as composite outcomes. RAS blockade users showed an increased risk of renal death in PS-matched analysis (hazard ratio [HR], 1.381; 95% CI, 1.071–1.781; *P* = 0.013), which was in agreement with the results of IPTW analysis (HR, 1.298; 95% CI, 1.123–1.500; *P* < 0.001). The risk of composite outcomes was higher in RAS blockade users in IPTW (HR, 1.154; 95% CI, 1.016–1.310; *P* = 0.027), but was marginal significance in PS matched analysis (HR, 1.243; 95% CI, 0.996–1.550; *P* = 0.054). The habitual use of RAS blockades in pre-dialysis patients with advanced CKD may have a detrimental effect on renal outcome without improving all-cause mortality. Further studies are warranted to determine whether withholding RAS blockade may lead to better outcomes in these patients.

## Introduction

The use of renin-angiotensin system (RAS) blockers such as angiotensin-converting enzyme inhibitors (ACEIs) and angiotensin receptor blockers (ARBs) are first-line options for reducing proteinuria and slowing the progression of nephropathy in diabetic patients. Moreover, RAS blockers are the antihypertensive drugs of choice in patients with non-diabetic chronic kidney disease (CKD) [1–4]. These recommendations are based on numerous reports that RAS blockers are more effective in slowing renal progression than other antihypertensive agents [5–11]. However, despite the use of RAS blockers to prevent the progression of CKD in the last two decades, the incidence of end-stage renal disease (ESRD) has continued to increase [12–15].

Although it is widely accepted that RAS blockades have specific renoprotective effects in CKD patients, the supporting evidence is not definitive. Indeed, a number of rigorous analyses of major studies have questioned the protective effects of RAS blockade, and noted several uncertainties [16–19]. In addition, the existence of blood pressure-independent beneficial effects of RAS blockades on renal outcome is controversial. Indeed, critical reviews and meta-analyses of studies on the renoprotective effects of ACEIs or ARBs could not dissociate these effects from the antihypertensive effects of RAS blockade, suggesting uncertainty in the benefits of ACEI/ARB for renal outcomes beyond reducing blood pressure [11, 17, 20–22]. However, other previous studies have reported positive results for RAS blockades, although they were not superior to other drugs in terms of reducing renal progression or the long-term risk of ESRD [23–25]. Thus, these findings raise a question about the advantage of ACEI/ARB in terms of renoprotection.

Most of large-scale clinical trials supporting the use of RAS blockades were principally conduced in populations comprising middle-aged individuals who had preserved renal function or mild to moderate renal insufficiency (CKD stage 1 to 3). Although there were previous studies that included severe renal insufficiency (CKD stage 4) [26, 27], not only they made up a small proportion of the published studies, but also pre-dialysis advanced CKD patients such as CKD stage 5 were mostly excluded. Therefore, it remains unclear if the renoprotective effects of RAS blockade also occur in patients with advanced CKD including pre-dialysis CKD.

There is uncertainty regarding the risks and benefits associated with the use of RAS blockade in patients with advanced CKD (stage 4 or 5). Therefore, this study assessed the effects of habitual use of RAS blockers on the risk of initiation of renal replacement therapy (RRT) and/or death and hospitalization.

## Materials and Methods

### Study design and participants

This was a retrospective propensity score (PS)-matched study on the effects of RAS blockers on renal outcomes and/or death in pre-dialysis patients with severe advanced CKD (stage 4 or 5). The data used were from adults aged ≥19 years who presented to one of four tertiary hospitals—Gachon University Gil Medical Center (Incheon, Korea), Cheju Halla General Hospital (Jeju, Korea), Chosun University Hospital (Gwangju, Korea), and Chungbuk National University Hospital (Cheongju, Korea)—with renal problems between November 1999 and December 2014. Initially, a total of 33,722 CKD patients were identified, and 3,239 subjects with stage 4 or 5 CKD (eGFR <30 mL/min/1.73m^2^ using the modification of diet in renal disease [MDRD] study equation) were selected [28]. From that group, 1,163 subjects who had received RRT prior to entry into the study (n = 23) and for which there was insufficient information about comorbidities or comparable laboratory data (n = 1,140) were excluded. Thus, a total of 2,076 advanced CKD patients were included in the analysis. This study was conducted with the approval of the institutional review board (IRB) of each of the four institutes (GCIRB2016-089, 2016-M09, 2016-08-004, and 2016-06-003-001) and performed in accordance with the principle of Helsinki Declaration. The IRBs waived the requirement for written informed consent because the study was of a retrospective observational design and did not involve interventions.

### Study variables

Demographic, clinical, and laboratory data were obtained by review of electronic medical records. The demographic and clinical data included age, sex, with or without nephrology care, medications, and medical comorbidities (diabetes, hypertension, and cardiovascular disease [CVD]). Diabetes and hypertension were identified using the validated ICD-10 codes. CVD was defined as angina pectoris, myocardial infarction, other ischemic heart disease, atrial fibrillation, heart failure, or cerebrovascular disease. Information about medications included antihypertensive medications including RAS blockers (ACEI/ARB), calcium-channel blockers (CCBs), beta-blockers, diuretics, and statins. The laboratory data included the magnitude of proteinuria and serum levels of creatinine, hemoglobin, albumin, calcium, and phosphorus. Proteinuria was measured by the dipstick test and was defined as negative, trace, or greater. GFR was estimated using the original four-variable MDRD equation as follows: eGFR = 186 × (serum creatinine)^-1.154^×(age)^-0.203^×0.742 (if female) [28].

### Outcome endpoints

The primary outcome of interest was the development of ESRD that required long-term dialysis, and the secondary endpoint was the composite outcome (ESRD, all-cause mortality, and hyperkalemia-associated hospitalization). The onset of ESRD was defined as the date of initiation of long-term dialysis (≥ 3 months), and the onset of the composite outcome was the date of initiation of long-term dialysis or death or hospitalization, whichever came first.

### Statistical analysis

For data description, continuous variables with a normal distribution were expressed as means ± standard deviation (SD), and categorical variables as frequencies and percentages. Continuous variables were compared using Student’s *t*-test, and categorical variables were compared using the χ^2^ test or Fisher’s exact test, as appropriate, prior to PS matching. To reduce the impact of selection bias and potential confounding factors due to differences in patient characteristics associated with treatment allocation in a non-randomized observational study, rigorous adjustment for differences in baseline characteristics was performed using inverse probability of treatment-weighted (IPTW) and PS-matched analyses. The PS of all of the subjects was estimated by modeling the probability of receiving RAS blockade. To determine the probability of receiving RAS blockade, a multivariable logistic model was constructed with the following covariates: age, sex, nephrologist visit, diabetes, hypertension, CVD, eGFR, proteinuria, serum levels of hemoglobin, albumin, calcium, phosphorus; and use of CCBs, beta blockers, diuretics, or statins. Using the multivariable logistic model, a PS was calculated for each individual. Subsequently, the derived PS values were used to match ACEI/ARB users with non-users at a ratio of 1:1 using the greedy matching algorithm. Following PS generation, weights of patients were calculated for IPTW analysis; weights for ACEI/ARB users were the inverse of those for PS and weights for non-users were the inverse of (1-PS). If the distribution of PS is highly variable, the treatment pattern will have extremely large weights [29]. Therefore, stabilized IPTWs were calculated to reduce the variability and ensure unbiased estimation of the treatment effect [29–32]. The discrimination and goodness of fit were assessed using the C-statistics and the Hosmer-Lemeshow test. After PS matching, the balance of covariates between the groups was assessed using the standardized differences. For the matched cohort, comparisons between ACEI/ARB users and non-users were performed using a paired *t*-test and McNemar test for continuous and categorical variables, respectively. The Kaplan-Meier method was applied to estimate the unadjusted cumulative incidence of primary and composite outcomes, and a log-rank test was used to assess differences between the groups. A Cox’s proportional hazard regression was performed to estimate adjusted hazard ratios (HRs) of ACEI/ARB use with 95% confidence intervals (CIs) for the incidence of ESRD and the composite outcome. The adjusted covariates used in the Cox regression were as follows: age, sex, nephrologist visit, diabetes, hypertension, CVD, eGFR, proteinuria, serum levels of hemoglobin, albumin, calcium, phosphorus; and use of CCB, beta-blockers, diuretics, or statins. Two-sided P values are reported, and a P value < 0.05 was considered statistically significant. PS matching was performed with the SAS software package (SAS Institute, version 9.3, Cary, NC); other analyses were performed using the SPSS Statistics software package (version 21.0, Chicago, IL).

## Results

### Study population and baseline characteristics

**Fig 1:**
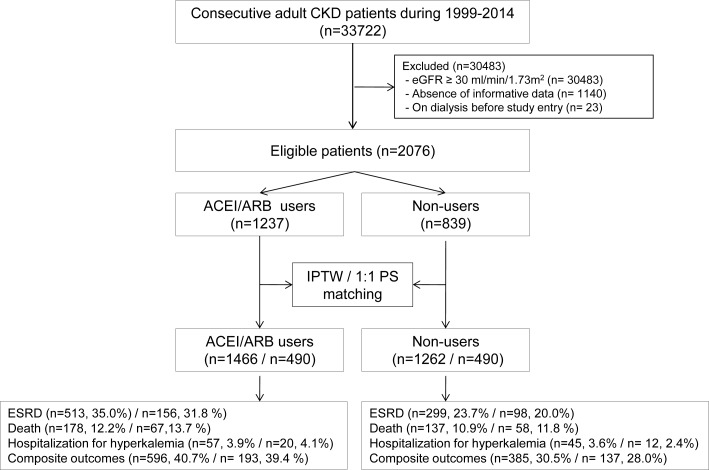
Flow chart of cohort formation. CKD, chronic kidney disease; eGFR, estimated glomerular filtration rate; ACEI, angiotensin converting enzyme inhibitor; ARB, angiotensin receptor blocker; IPTW, inverse probability of treatment weighted; PS, propensity score; ESRD, end stage renal disease.

A total of 2,076 patients with advanced CKD (stage 4 or 5) met the inclusion criteria and were included in the analyses. The demographic and clinical characteristics of the study population are shown in Table 1. Among the subjects, 1,237 (59.6%) were prescribed an ACEI/ARB and 14.2% (n = 176) of ACEI/ARB users were treated with combination of ACEI and ARB. The average age of ACEI/ARB users was 60.5 ± 15.1 years, and 45.9% were females. Compared to non-users, ACEI/ARB users were more likely to have medical comorbidities such as diabetes and CVD, and exhibited greater use of other antihypertensive drugs including CCBs, beta-blockers, and diuretics. Statins were also prescribed more frequently to ACEI/ARB users than non-users. Non-users of ACEI/ARB were older, less likely to visit a nephrologist, and had a lower eGFR compared to users. Serum levels of hemoglobin, albumin, and calcium were not significantly different between the two groups. Using PS estimation methods, IPTW and PS matching analyses were performed (Fig 1); the results indicated that the baseline characteristics of the ACEI/ARB users and non-users were not significantly different (Table 1). Among 490 ACEI/ARB users, 63 patients (14.8%) were on dual treatment of ACEI and ARB.

**Table 1:** Clinical characteristics of study participants.

		Original data			IPTW data			PS matching data			
		ACEI/ARB user	ACEI/ARB non-user	*P*	ACEI/ARB user	ACEI/ARB non-user	*P*	ACEI/ARB user	ACEI/ARB non-user	*P*	Standardized differences
		n = 1,237	n = 839		n = 1,466	n = 1,262		n = 490	n = 490		
Age, year		60.5±15.1	61.9±15.1	0.041	61.0±15.2	60.7±15.7	0.730	60.1±15.9	60.5±15.6	0.648	0.035
Female gender, n (%)		568 (45.9%)	422 (50.3%)	0.050	691 (47.2%)	589 (46.7%)	0.796	223 (45.5%)	242 (49.4%)	0.248	0.094
Nephrologist visit, n (%)		870 (70.3%)	497 (59.2%)	<0.001	992 (67.7%)	837 (66.3%)	0.439	321 (65.5%)	331 (67.6%)	0.529	0.045
Diabetes, n (%)		626 (50.6%)	228 (27.2%)	<0.001	631 (43.0%)	511 (40.5%)	0.178	186 (38.0%)	183 (37.3%)	0.889	0.014
Hypertension, n (%)		910 (73.6%)	259 (30.9%)	<0.001	855 (58.3%)	715 (56.7%)	0.380	237 (48.4%)	241 (49.2%)	0.803	0.016
Previous CVD, n (%)		366 (29.6%)	109 (13.0%)	<0.001	346 (23.6%)	302 (23.9%)	0.848	90 (18.4%)	84 (17.1%)	0.675	0.034
eGFR, ml/min/1.73m^2^		17.8±7.7	15.4±7.7	<0.001	16.9±7.8	16.3±7.6	0.038	16.2±7.7	16.4±7.5	0.699	0.029
Proteinuria, n (%)		1,121 (90.6%)	746 (88.9%)	0.205	1,318 (89.9%)	1,150 (91.1%)	0.279	440 (89.9%)	438 (89.4%)	0.918	0.048
	Negative	116 (9.4%)	93 (11.1%)		148 (10.1%)	112 (8.9%)		50 (10.2%)	52 (10.6%)		0.013
	Trace (±)	115 (9.3%)	73 (8.7%)		138 (9.4%)	102 (8.1%)		43 (8.8%)	45 (9.2%)		0.014
	(+)	188 (15.2%)	174 (20.7%)		260 (17.7%)	249 (19.7%)		81 (16.5%)	99 (20.2%)		0.096
	(++)	317 (25.6%)	250 (29.8%)		371(25.3%)	372 (29.5%)		134 (27.3%)	143 (29.2%)		0.042
	(+++)	376 (30.5%)	187 (22.3%)		405 (27.7%)	309 (24.5%)		127 (25.9%)	111 (22.7%)		0.075
	(++++)	125 (10.1%)	62 (7.4%)		142 (9.7%)	118 (9.4%)		55 (11.2%)	40 (8.2%)		0.101
Hemoglobin, g/dl		10.2±2.2	10.1±2.3	0.317	10.2±2.2	10.1±2.1	0.610	10.0±2.2	10.2±2.2	0.236	0.059
Albumin, g/dl		3.5±0.7	3.5±0.7	0.510	3.5±0.7	3.6±0.7	0.547	3.5±0.7	3.5±0.7	0.848	0.006
Calcium, mg/dl		8.5±0.9	8.5±1.0	0.595	8.5±0.9	8.6±1.0	0.481	8.5±1.0	8.5±1.0	0.786	0.008
Phosphorus, mg/dl		4.4±1.5	4.6±1.9	0.011	4.5±1.6	4.6±1.8	0.527	4.6±1.7	4.6±1.8	0.779	0.016
Beta-blockers, n (%)		803 (64.9%))	235 (28.0%)	<0.001	778 (53.1%)	661 (52.4%)	0.718	227 (46.3%)	220 (44.9%)	0.654	0.025
CCB, n (%)		941 (76.1%))	298 (35.5%)	<0.001	908 (62.0%)	769 (60.9%)	0.576	272 (55.5%)	274 (55.9%)	0.938	0.028
Diuretics, n (%)		929 (75.1%)	308 (36.7%)	<0.001	906 (61.8%)	760 (60.2%)	0.387	291 (59.4%)	283 (57.8%)	0.589	0.008
Statin, n (%)		517 (41.8%)	103 (12.3%)	<0.001	450 (30.7%)	370 (29.3%)	0.419	94 (19.2%)	99 (20.2%)	0.714	0.032

Continuous data are presented as the mean ± SD and categorical data are presented as number (percentages). IPTW, inverse probability of treatment weighted; PS, propensity score; ACEI, angiotensin converting enzyme inhibitor; ARB, angiotensin receptor blocker; CVD, cardiovascular disease; eGFR, estimated glomerular filtration rate; CCB, calcium channel blocker.

### ESRD and composite outcome in the overall cohort

**Fig 2:**
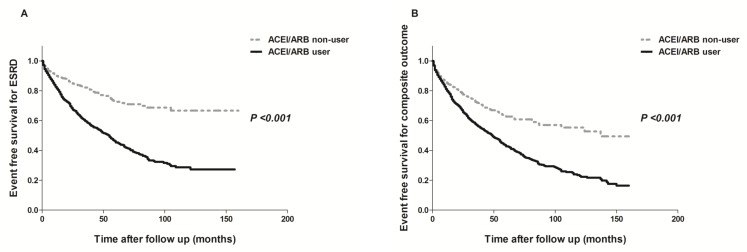
**Kaplan-Meier curves for ESRD requiring RRT (A) and composite outcome (ESRD or all-cause mortality or hospitalization for hyperkalemia) (B) in overall patient cohort.** ACEI/ARB users showed higher risk of renal mortality (A) and composite outcome (B) than non-users.

Median follow-up times for ESRD and death were 16 months (interquartile range, 3.0–44.0 months) and 28 months (interquartile range, 6.0–62.0 months), respectively. During the observation period, a total of 631 (30.4%) patients developed ESRD requiring long-term RRT, and 257 (12.4%) patients died. ACEI/ARB users had a significantly higher risk of developing ESRD compared to non-users (*P* < 0.001; Fig 2). All-cause mortality was not significantly different between the two groups (*P* = 0.075). However, the rate of hospitalization for hyperkalemia was higher in ACEI/ARB users than in non-users (*P* = 0.042). In addition, use of ACEI/ARB was significantly associated with a greater risk of developing the composite outcome of ESRD or death from any cause or hospitalization for hyperkalemia (*P* < 0.001; Fig 2). The adjusted HR for ESRD (HR, 1.383; 95% CI, 1.107–1.729; *P* = 0.004) was significantly higher in ACEI/ARB users compared to non-users, but the adjusted HR for the composite outcome was not different between the two groups (HR, 1.180; 95% CI, 0.980–1.420; *P* = 0.080; Table 2). When analysis was performed among the three groups (non-users, ACEI or ARB users, and ACEI+ARB users), ACEI or ARB users showed consistent results showing the significantly increased HRs for ESRD and composite outcome. However, the increased HRs for ESRD and composite outcomes were not significant between non-users and ACEI+ARB users (S1–S3 Tables).

**Table 2:** Hazard ratios for clinical outcomes according to analytic method comparing ACEI/ARB user vs. non-user.

	ESRD		All-cause mortality		Composite outcome	
	HR (95% CI)	*P* value	HR (95% CI)	*P* value	HR (95% CI)	*P* value
Univariate Cox Model (n = 2,076)	2.214 (1.807–2.711)	<0.001	0.791 (0.610–1.025)	0.076	1.646 (1.396–1.940)	<0.001
Multivariate Cox Model^a^ (n = 2,076)	1.383 (1.107–1.729)	0.004	0.827 (0.607–1.126)	0.228	1.180 (0.980–1.420)	0.080
Inverse probability of treatment weighting^a^ (n = 2,728)	1.298 (1.123–1.500)	<0.001	0.826 (0.659–1.035)	0.097	1.154 (1.016–1.310)	0.027
Propensity score matching^a^ (n = 980)	1.381 (1.071–1.781)	0.013	0.874 (0.609–1.255)	0.466	1.243 (0.996–1.550)	0.054

^a^ Adjusted for age, sex, nephrologist visit, diabetes, hypertension, cardiovascular disease, estimated glomerular filtration rate, proteinuria, serum hemoglobin, albumin, calcium, phosphours, use of beta-blocker, calcium channel blocker, diuretics, statin.

ESRD, end stage renal disease; HR, hazard ratio; 95% CI, 95% confidential interval.

### ESRD and the composite outcome in matched cohort

**Fig 3:**
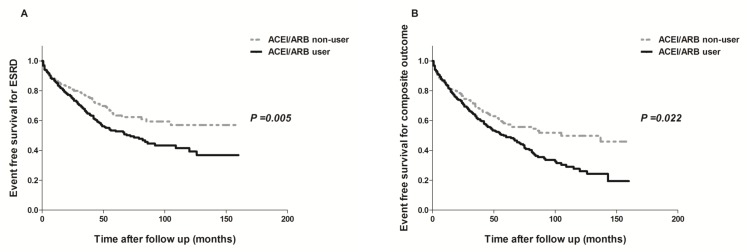
**Kaplan-Meier curves for ESRD requiring RRT (A) and composite outcome (ESRD or all-cause mortality or hospitalization for hyperkalemia) (B) in propensity score matching cohort.** ACEI/ARB users showed higher risk of renal mortality (A) and composite outcome (B) than non-users.

In the PS-matched cohort, the risk of developing ESRD was significantly higher in ACEI/ARB users than in non-users (P = 0.005; Fig 3). The rates of all-cause mortality and hospitalization for hyperkalemia were not significantly different between the two groups (*P* = 0.837 and *P* = 0.302). However, the risk of composite outcome was significantly increased in ACEI/ARB users compared to non-users (P = 0.022; Fig 3). The adjusted HR for outcomes together with the results of PS matching and IPTW analyses are shown in Table 2. In the PS-matched analysis, the adjusted HR for ESRD was significantly higher in ACEI/ARB users than in non-users (HR, 1.381; 95% CI, 1.071–1.781; *P* = 0.013), which was consistent with the findings following IPTW adjustment (HR, 1.298; 95% CI, 1.123–1.500; *P* < 0.001). The adjusted HR for the composite outcome of ESRD or death from any cause or hospitalization for hyperkalemia was higher in ACEI/ARB users than in non-users, but did not reach statistically significant levels (HR, 1.243; 95% CI, 0.996–1.550; *P* = 0.054) in the PS-matched analysis. However, IPTW adjustment resulted in a significantly increased risk of composite outcome (HR, 1.5495% CI, 1.016–1.310; *P* = 0.027). Additional analysis performed among the three groups (non-users, ACEI or ARB users, and ACEI+ARB users), ACEI or ARB users showed similar patterns, but ACEI+ARB users did not show significant different outcomes compared with non-users (S1–S3 Tables). Furthermore, in the stratified analyses by doses of RAS blockers, the tendency to increase risks of ESRD and composite outcome were observed in both low-dose and high-dose ACEI/ARB users as well, although the results could not reach the statistical significance (data not shown).

### Subgroup analyses of outcomes in the matched cohort

**Fig 4:**
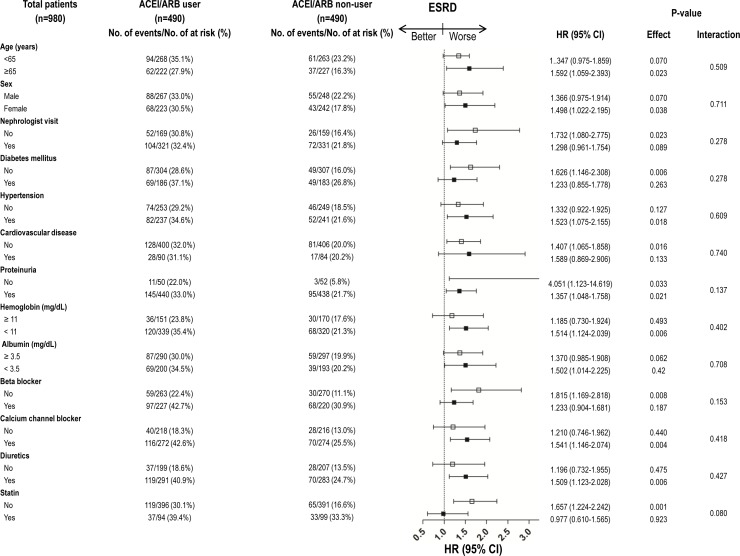
Subgroup analyses comparing hazard ratios (HRs) for ESRD requiring RRT between ACEI/ARB user and ACEI/ARB non-user in propensity score matching cohort.

**Fig 5:**
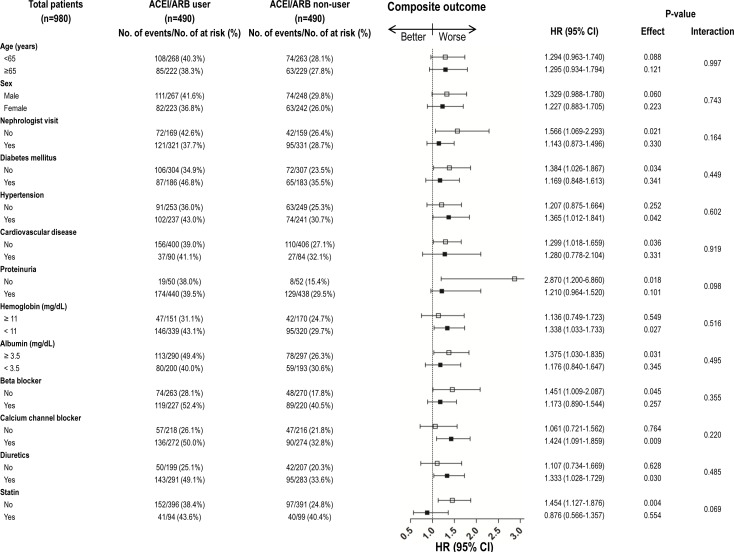
Subgroup analyses comparing hazard ratios (HRs) for composite outcome (ESRD or all-cause mortality or hospitalization for hyperkalemia) between ACEI/ARB user and ACEI/ARB non-user in propensity score matching cohort.

Stratified analyses of ESRD and the composite outcome according to patient characteristics were conducted in the matched cohort (Figs 4 and 5). The increased HRs for ESRD and the composite outcome, indicative of a worse outcome in ACEI/ARB users, were consistent across the majority of patient subgroups.

## Discussion

In this study, which was conducted in pre-dialysis patients with advanced CKD (stages 4 and 5), the use of ACEI/ARB was associated with an increased risk of developing ESRD, necessitating long-term dialysis and the composite outcome of ESRD or death from any cause, or hospitalization for hyperkalemia. After controlling for potential confounding factors using PS matching and IPTW, the findings suggested an increased risk of ESRD, but no difference in all-cause mortality, in ACEI/ARB users compared to non-users. This suggests that the use of RAS blockers in this patient population may accelerate progression to ESRD without enhancing survival.

Our findings are not in agreement with the pre-existing belief that the use of RAS blockers has favorable effects on renal outcomes. Numerous studies have shown that RAS blockade using an ACEI or ARB slowed the rate of renal progression [5–11], which has led to the increasing use of RAS blockade in CKD patients. Therefore, evidence supporting the renoprotective effects of RAS blockade should be carefully reconsidered. The first issue is the benefit of RAS blockade beyond reducing blood pressure. Several studies have questioned the blood-pressure-independent renoprotective effects of RAS blockade, as the benefit of RAS blockade on renal outcomes could not be dissociated from its blood pressure-lowering effect [11, 16, 17, 20–22]. Indeed, in the Heart Outcomes Prevention Evaluation (HOPE) substudy, ambulatory blood pressure was significantly lower in the Ramipril treatment group than the placebo group, suggesting that the benefits of ACEI can be attributed to their blood pressure-lowering effects [20]. Second, some studies have failed to show the beneficial effects of RAS blockade or have reported the reverse outcomes. In the UK prospective diabetes study on patients with hypertension and type 2 diabetes, the incidence of renal failure was not different between the captopril and atenolol groups, and both groups had a similar reduction in blood pressure [33]. Suissa *et al*. [25] reported an increased risk of ESRD in a population-based cohort of diabetic patients who persistently took ACEI. Moreover, combination treatments of ACEI and ARB worsened renal outcomes—including dialysis, doubling of serum creatinine, and death—in subjects with a high cardiovascular risk [34]. Third, most studies that reported results favoring use of RAS blockades involved middle-aged patients with relatively well-preserved renal function and few complications. Moreover, few studies included advanced CKD patients including CKD stage 4 or 5. Baseline kidney function is an important factor for renal outcomes; therefore, whether the renoprotective effects of RAS blockades in early CKD patients would also occur in advanced CKD patients is unclear. Indeed, several studies conducted in patients with advanced renal insufficiency have reported results different from the general consensus on RAS blockade in those patients. A small observational study demonstrated that the discontinuation of ACEI/ARB in advanced CKD (stage 4 or 5) patients led to an overall improvement in eGFR and delayed the initiation of RRT [35]. Similar findings in advanced CKD elderly patients were reported by Qnuibgo *et al*. [36, 37].

Hou *et al*. [27] demonstrated the use of ACEI significantly improved renal outcome in patients with non-diabetic CKD stage 4, which was not in agreement with our findings. However, there were several differences in study population between their work and our study. Our study population had more severe renal insufficiency (mean eGFR; 18 mL/min/1.73m^2^) than those of the study (mean eGFR; 26 mL/min/1.73m^2^), because we included patients with CKD stage 5 (42.3%) as well as CKD stage 4 (57.3%). Moreover, we included diabetic patients while they included only non-diabetic patients. In addition, recently, Hsu *et al*. [38] conducted a population-based study involving pre-dialysis CKD patients with anemia who had received an erythropoiesis-stimulating agent (ESA). The use of ACEI/ARB was associated with a reduced risk of long-term dialysis, which was inconsistent with our findings. However, the authors did not report biochemical parameters, which could be important prognostic factors for renal outcomes in these patients. In addition, the study was limited to pre-dialysis CKD patients with anemia undergoing ESA treatment. According to published data, the advanced CKD population includes a considerable number of patients without anemia, despite the fact that the prevalence of anemia increases with declining renal function (the prevalence of anemia is 30–50% in patients with stage 4 or 5 CKD) [39–41]. Thus, the effectiveness of RAS blockade cannot be generalized to all pre-dialysis patients with advanced CKD. In comparison, this study included pre-dialysis patients with and without anemia, and analyzed relevant laboratory findings to control for potential confounding factors. These differences might have contributed to the discrepant results between the two studies.

The mechanisms underlying the association of RAS blockade with an increased risk of renal failure in advanced CKD patients is unclear. Possibilities include the fact that the prevalence of CKD increases with age, and all of the stages of CKD are more prevalent at older ages [42, 43]. Thus, the majority of advanced CKD patients are likely to be elderly people who are susceptible to the nephrotoxic effects of drugs (including ACEI/ARBs), and are less likely to recover their kidney function following iatrogenic renal injury [44, 45]. In addition, it is possible that microvascular renal arteriolar stenosis is undiagnosed in advanced CKD patients, and the use of ACEI/ARBs in patients with renal arteriolar stenotic lesions might exacerbate the decline in GFR, mimicking renal artery stenosis. Indeed, these renal microvascular lesions are more prevalent in the elderly [36, 46]. In previous reports, consistent renoprotective effects of RAS blockades were evident in patients with significant proteinuria [47–50]. In other words, the beneficial effects of RAS blockade were limited to a highly selective population, in whom renal insufficiency was associated with heavy proteinuria due to pure glomerular disease. These selective effects of RAS blockade explain the lack of a beneficial effect in this study population, which comprised patients with non-glomerular ischemic CKD as well as those with pure glomerular disease. In our research, contrary to the harmful effect on ESRD, the use of RAS blocker showed a beneficial tendency on all-cause mortality, even though it was not statistically significant. Among previous studies regarding the effect of RAS blocker, several studies investigated the efficacy of RAS blocker on all-cause mortality comparing ACEI and ARB. Some studies reported heterogeneity for all-cause mortality between ACEI and ARB showing the priority of ACEI to ARB in improving survival [51, 52], while there were reports showing that the outcome with ACEI was similar to those with ARB [53, 54]. In our study, the stratified analysis according to the class of drug (ACEI and ARB) showed decreased HR for all-cause mortality in ARB users but increased HR in ACEI users, even though both HRs were not statistically significant, whereas significant increased risks of ESRD were observed in both ACEI and ARB users (S4–S6 Tables). Therefore, the observed beneficial tendency on all-cause mortality may be attributed to the possible differential effects of ACEI and ARB on all-cause mortality.

This study investigated the effects of RAS blockade in pre-dialysis patients with advanced CKD, who had been excluded from the majority of previous studies. Moreover, a relatively large number of patients with stage 4 or 5 CKD at multiple centers was included. However, this study had several limitations. First, this was an observational study; thus, it had the inherent drawback of random allocation to a treatment group (users of RAS blockers) or non-treatment group (non-users of RAS blockers), which could lead to selection bias. To reduce such bias and minimize differences in the baseline characteristics between the two groups, IPTW and PS-matched analyses were performed. However, this is an imperfect substitute for a randomized study and can result in hidden bias due to differences in unmeasured covariates. In addition, the causality of the results could not be inferred due to the study design. Second, the present study is not feasible to assess the change in blood pressure and proteinuria according treatment due to an uncontrolled study design, but blood pressure and proteinuria are known to be important risk factors for renal outcome. The change in blood pressure and proteinuria might contribute to confounding effects on the use of RAS blocker and renal outcome. Third, previous studies have shown that adding mineralocorticoid receptor antagonist (MRA) to ACEI and/or ARB reduced in proteinuria and blood pressure, even though whether it prevents renal progression is unknown [55, 56]. In addition, there were some reports on the effects of the other RAS blocker such as direct renin inhibitor [57, 58]. However, there was a lack of information on the concurrent use of MRA or direct renin inhibitor that could exert an influence on our study outcomes. Lastly, whether discontinuation of RAS blockade resulted in improvement of renal function and slowing of renal progression in patients with advanced CKD could not be determined because this was not an interventional study. In summary, the habitual use of RAS blockades in patients with stage 4 or 5 advanced CKD was associated with an increased risk of ERSD requiring RRT. In other words, the renoprotective effects of RAS blockades in these patients should be carefully reconsidered. Moreover, the composite clinical outcome of ESRD or death from any cause or hospitalization for hyperkalemia was more prevalent in users than non-users of RAS blockers.

In conclusion, ACEI/ARB treatment in pre-dialysis patients with advanced CKD may have detrimental effects on renal outcome without improving all-cause mortality. Further studies are warranted to determine whether withholding ACEI/ARB treatment leads to improved outcomes.

## Supporting Information

S1 Table Hazard ratios for ESRD according to analytic method comparing ACEI or ARB users vs. non-users and ACEI+ARB users vs. non-users. (DOCX)

S2 Table Hazard ratios for Death according to analytic method comparing ACEI or ARB users vs. non-users and ACEI+ARB users vs. non-users. (DOCX)

S3 Table Hazard ratios for Composite outcome according to analytic method comparing ACEI or ARB users vs. non-users and ACEI+ARB users vs. non-users. (DOCX)

S4 Table Hazard ratios for ESRD according to analytic method comparing ARB users vs. non-users and ACEI users vs. non-users. (DOCX)

S5 Table Hazard ratios for Death according to analytic method comparing ARB users vs. non-users and ACEI users vs. non-users. (DOCX)

S6 Table Hazard ratios for Composite outcome according to analytic method comparing ARB users vs. non-users and ACEI users vs. non-users. (DOCX)
