# Supplementary material for: The Impact of Renin-Angiotensin System Blockade on Renal Outcomes and Mortality in Pre-Dialysis Patients with Advanced Chronic Kidney Disease
Source: PLoS One. 2017 Jan 25;12(1):e0170874. doi: 10.1371/journal.pone.0170874 (PMC5266335; doi:10.1371/journal.pone.0170874)
Supplement: S1 Table — (DOCX) [file pone.0170874.s001.docx]

**S1 Table.** **Hazard ratios for ESRD according to analytic method comparing ACEI or ARB users vs. non-users and ACEI+ARB users vs. non-users**

|  | Non-user | | ACEI or ARB user | | ACEI +ARB user | |
| --- | --- | --- | --- | --- | --- | --- |
|  | HR (95% CI) | *P* value | HR (95% CI) | *P* value | HR (95% CI) | *P* value |
| Univariate Cox Model (n=2,076) | 1.00 | reference | 2.239 (1.821-2.753) | <0.001 | 2.160 (1.636-2.852) | <0.001 |
| Multivariate Cox Model^a^ (n=2,076) | 1.00 | reference | 1.434 (1.145-1.796) | 0.002 | 1.034 (0.738-1.450) | 0.846 |
| Inverse probability of treatment weighting^a^ (n=2,728) | 1.00 | reference | 1.390 (1.190-1.624) | <0.001 | 0.973 (0.764-1.240) | 0.826 |
| Propensity score matching^a^ (n=980) | 1.00 | reference | 1.469 (1.126-1.917) | 0.005 | 1.092 (0.699-1.7070) | 0.699 |

^a^ Adjusted for age, sex, nephrologist visit, diabetes, hypertension, cardiovascular disease, estimated glomerular filtration rate, proteinuria, serum hemoglobin, albumin, calcium, phosphours, use of beta-blocker, calcium channel blocker, diuretics, statin.

ESRD, end stage renal disease; HR, hazard ratio; 95% CI, 95% confidential interval.
