# Supplementary material for: The Impact of Renin-Angiotensin System Blockade on Renal Outcomes and Mortality in Pre-Dialysis Patients with Advanced Chronic Kidney Disease
Source: PLoS One. 2017 Jan 25;12(1):e0170874. doi: 10.1371/journal.pone.0170874 (PMC5266335; doi:10.1371/journal.pone.0170874)
Supplement: S2 Table — (DOCX) [file pone.0170874.s002.docx]

**S2 Table.** **Hazard ratios for Death according to analytic method comparing ACEI or ARB users vs. non-users and ACEI+ARB users vs. non-users**

|  | Non-user | | ARB or ACEI user | | ACEI +ARB user | |
| --- | --- | --- | --- | --- | --- | --- |
|  | HR (95% CI) | *P* value | HR (95% CI) | *P* value | HR (95% CI) | *P* value |
| Univariate Cox Model (n=2,076) | 1.00 | reference | 0.873 (0.669-1.139) | 0.315 | 0.566 (0.357-0.898) | 0.016 |
| Multivariate Cox Model^a^ (n=2,076) | 1.00 | reference | 0.883 (0.648-1.205) | 0.433 | 0.687 (0.390-1.208) | 0.193 |
| Inverse probability of treatment weighting^a^ (n=2,728) | 1.00 | reference | 0.879 (0.693-1.115) | 0.287 | 0.420 (0.283-0.624) | <0.001 |
| Propensity score matching^a^ (n=980) | 1.00 | reference | 0.921 (0.630-1.345) | 0.670 | 0.839 (0.427-1.648) | 0.610 |

^a^ Adjusted for age, sex, nephrologist visit, diabetes, hypertension, cardiovascular disease, estimated glomerular filtration rate, proteinuria, serum hemoglobin, albumin, calcium, phosphours, use of beta-blocker, calcium channel blocker, diuretics, statin.

ESRD, end stage renal disease; HR, hazard ratio; 95% CI, 95% confidential interval.
