# Supplementary material for: The Impact of Renin-Angiotensin System Blockade on Renal Outcomes and Mortality in Pre-Dialysis Patients with Advanced Chronic Kidney Disease
Source: PLoS One. 2017 Jan 25;12(1):e0170874. doi: 10.1371/journal.pone.0170874 (PMC5266335; doi:10.1371/journal.pone.0170874)
Supplement: S3 Table — (DOCX) [file pone.0170874.s003.docx]

**S3 Table.** **Hazard ratios for Composite outcome according to analytic method comparing ACEI or ARB users vs. non-users and ACEI+ARB users vs. non-users**

|  | Non-user | | ARB or ACEI user | | ACEI +ARB user | |
| --- | --- | --- | --- | --- | --- | --- |
|  | HR (95% CI) | *P* value | HR (95% CI) | *P* value | HR (95% CI) | *P* value |
| Univariate Cox Model (n=2,076) | 1.00 | reference | 1.664 (1.406-1.970) | <0.001 | 1.619 (1.273-2.060) | <0.001 |
| Multivariate Cox Model^a^ (n=2,076) | 1.00 | reference | 1.204 (0.998-1.453) | 0.052 | 1.090 (0.809-1.468) | 0.571 |
| Inverse probability of treatment weighting^a^ (n=2,728) | 1.00 | reference | 1.206 (1.052-1.381) | 0.007 | 0.869 (0.706-1.071) | 0.188 |
| Propensity score matching^a^ (n=980) | 1.00 | reference | 1.250 (0.989-1.579) | 0.062 | 1.244 (0.855-1.810) | 0.254 |

^a^ Adjusted for age, sex, nephrologist visit, diabetes, hypertension, cardiovascular disease, estimated glomerular filtration rate, proteinuria, serum hemoglobin, albumin, calcium, phosphours, use of beta-blocker, calcium channel blocker, diuretics, statin.

ESRD, end stage renal disease; HR, hazard ratio; 95% CI, 95% confidential interval.
