# Supplementary material for: The Impact of Renin-Angiotensin System Blockade on Renal Outcomes and Mortality in Pre-Dialysis Patients with Advanced Chronic Kidney Disease
Source: PLoS One. 2017 Jan 25;12(1):e0170874. doi: 10.1371/journal.pone.0170874 (PMC5266335; doi:10.1371/journal.pone.0170874)
Supplement: S4 Table — (DOCX) [file pone.0170874.s004.docx]

**S4 Table.** **Hazard ratios for ESRD according to analytic method comparing ARB users vs. non-users and ACEI users vs. non-users**

|  | Non-user | | ARB user | | ACEI user | |
| --- | --- | --- | --- | --- | --- | --- |
|  | HR (95% CI) | *P* value | HR (95% CI) | *P* value | HR (95% CI) | *P* value |
| Univariate Cox Model (n=2,076) | 1.00 | reference | 2.267 (1.812-2.836) | <0.001 | 2.207 (1.750-2.783) | <0.001 |
| Multivariate Cox Model^a^ (n=2,076) | 1.00 | reference | 1.617 (1.216-2.151) | 0.001 | 1.366 (1.060-1.759) | 0.016 |
| Inverse probability of treatment weighting^a^ (n=2,728) | 1.00 | reference | 1.640 (1.363-1.973) | <0.001 | 1.411 (1.152-1.729) | 0.001 |
| Propensity score matching^a^ (n=980) | 1.00 | reference | 1.650 (1.141-2.387) | 0.008 | 1.514 (1.098)-2.090 | 0.011 |

^a^ Adjusted for age, sex, nephrologist visit, diabetes, hypertension, cardiovascular disease, estimated glomerular filtration rate, proteinuria, serum hemoglobin, albumin, calcium, phosphours, use of beta-blocker, calcium channel blocker, diuretics, statin.

ESRD, end stage renal disease; HR, hazard ratio; 95% CI, 95% confidential interval.
