# Supplementary material for: The Impact of Renin-Angiotensin System Blockade on Renal Outcomes and Mortality in Pre-Dialysis Patients with Advanced Chronic Kidney Disease
Source: PLoS One. 2017 Jan 25;12(1):e0170874. doi: 10.1371/journal.pone.0170874 (PMC5266335; doi:10.1371/journal.pone.0170874)
Supplement: S5 Table — (DOCX) [file pone.0170874.s005.docx]

**S5 Table.** **Hazard ratios for Death according to analytic method comparing ARB users vs. non-users and ACEI users vs. non-users**

|  | Non-user | | ARB user | | ACEI user | |
| --- | --- | --- | --- | --- | --- | --- |
|  | HR (95% CI) | *P* value | HR (95% CI) | *P* value | HR (95% CI) | *P* value |
| Univariate Cox Model (n=2,076) | 1.00 | reference | 0.708 (0.511-0.981) | 0.038 | 1.075 (0.794-1.455) | 0.640 |
| Multivariate Cox Model^a^ (n=2,076) | 1.00 | reference | 0.818 (0.536-1.250) | 0.354 | 1.002 (0.709-1.416) | 0.989 |
| Inverse probability of treatment weighting^a^ (n=2,728) | 1.00 | reference | 0.697 (0.522-0.931) | 0.015 | 0.939 (0.702-1.256) | 0.672 |
| Propensity score matching^a^ (n=980) | 1.00 | reference | 0.742 (0.411-1.339) | 0.321 | 1.104 (0.707-1.723) | 0.663 |

^a^ Adjusted for age, sex, nephrologist visit, diabetes, hypertension, cardiovascular disease, estimated glomerular filtration rate, proteinuria, serum hemoglobin, albumin, calcium, phosphours, use of beta-blocker, calcium channel blocker, diuretics, statin.

ESRD, end stage renal disease; HR, hazard ratio; 95% CI, 95% confidential interval.
