# Supplementary material for: The Impact of Renin-Angiotensin System Blockade on Renal Outcomes and Mortality in Pre-Dialysis Patients with Advanced Chronic Kidney Disease
Source: PLoS One. 2017 Jan 25;12(1):e0170874. doi: 10.1371/journal.pone.0170874 (PMC5266335; doi:10.1371/journal.pone.0170874)
Supplement: S6 Table — (DOCX) [file pone.0170874.s006.docx]

**S6 Table.** **Hazard ratios for Composite outcome according to analytic method comparing ARB users vs. non-users and ACEI users vs. non-users**

|  | Non-user | | ARB user | | ACEI user | |
| --- | --- | --- | --- | --- | --- | --- |
|  | HR (95% CI) | *P* value | HR (95% CI) | *P* value | HR (95% CI) | *P* value |
| Univariate Cox Model (n=2,076) | 1.00 | reference | 1.683 (1.396-2.029) | <0.001 | 1.651 (1.360-2.004) | <0.001 |
| Multivariate Cox Model^a^ (n=2,076) | 1.00 | reference | 1.375 (1.082-1.747) | 0.009 | 1.182 (0.953-1.465) | 0.128 |
| Inverse probability of treatment weighting^a^ (n=2,728) | 1.00 | reference | 1.267 (1.079-1.487) | 0.004 | 1.196 (1.003-1.426) | 0.046 |
| Propensity score matching^a^ (n=980) | 1.00 | reference | 1.318 (0.949-1.830) | 0.100 | 1.310 (0.986-1.740) | 0.063 |

^a^ Adjusted for age, sex, nephrologist visit, diabetes, hypertension, cardiovascular disease, estimated glomerular filtration rate, proteinuria, serum hemoglobin, albumin, calcium, phosphours, use of beta-blocker, calcium channel blocker, diuretics, statin.

ESRD, end stage renal disease; HR, hazard ratio; 95% CI, 95% confidential interval.
